# Life Cycle Assessment (LCA) of Substrate Mixes Containing Port Sediments for Sustainable ‘Verna’ Lemon Production

**DOI:** 10.3390/foods11193053

**Published:** 2022-10-01

**Authors:** Francisca Hernández, Juan José Martínez-Nicolás, Pablo Melgarejo, Dámaris Núñez-Gómez, Vicente Lidón, Rafael Martínez-Font, Pilar Legua

**Affiliations:** Centro de Investigación e Innovación Agroalimentaria y Agroambiental (CIAGRO-UMH), Miguel Hernández University, Ctra. Beniel, km 3.2, 03312 Orihuela, Alicante, Spain

**Keywords:** dredged port sediment, life cycle assessment, *Citrus limon* L. Burm, circular economy, waste revalorization

## Abstract

The increase in maritime trade and its global economic importance have forced port management actors to carry out the periodic dredging of their sediments to maintain an adequate depth for the passage of large ships to maintain their operation and competitiveness. During the dredging process, large volumes of port sediment are generated. Dredged port sediment is currently considered a waste material and its disposal is regulated. Finding ways to safely reuse port sediments is necessary for sustainable development. In this study, a life cycle assessment (LCA) methodology was applied to identify the environmental impact of port sediments when used as a culture medium for lemon trees. A total of 90 lemon trees (*Citrus limon* L. Burm var ‘Verna’) were used in the trial. The trees were grown under controlled conditions using three substrates, with different portions of peat and port sediment (25%, 50%, and 75%) to identify the real impacts of the culture media on the growth process. The LCA was calculated and analyzed according to the ISO 14040:2006 standard, using the SimaPro v. 9.3 software (PRé Sustainability B.V, Amersfoort, The Netherlands). The functional unit defined for the three-culture media was 1 kg of lemons. The LCA results showed a significant increase in the environmental impact of lemon cultivation proportional to port sediment content (75%), due to the decrease in fruit production caused by the sediment. However, the least impact was identified for the culture medium at 50% peat and 50% port sediment. The greatest impacts were more related to crop management rather than the port sediment content. The results showed that the use of the port sediment, mixed with other substrates as an agricultural medium amendment, is a viable option for lemon growers.

## 1. Introduction

More than 8000 ports exist in 222 countries. The smooth and continuous operation of shipping is essential for the exchange of food, energy, raw materials, and manufactured products all over the world. Between 2018 and 2019, port activity was responsible for more than 70% of the volume of world trade due to both the improvement in port management and the increase in the size of ships [1]. This increase in the size of merchant ships requires deeper waterways; therefore, commercial ports are forced to increase the depth of their channels on a regular basis to maintain their operation and improve their competitiveness [2], so large volumes of dredged port sediment are generated each year.

Due to its physical, chemical, and compositional characteristics (metal ions and hydrocarbons content, among other pollutants derived from port activities), dredged port sediments are considered waste, so current legislation regulates their reuse and direct disposal in appropriate industrial landfills [3,4,5]. Over recent years, there has been a significant increase in studies focused on sustainable alternatives for dredged port sediment management, many of which focus on its reuse as an alternative cultivation substrate to peat [4,6,7,8,9,10]. The forecast depletion, environmental impact, and rising prices of the substrates commonly used in agriculture, such as peat and/or coconut fiber, forced growers and suppliers to look for alternative substrates that can substitute and/or reduce their demand without compromising the vegetative development of plantations and by maximizing crop production [11,12].

For example, since 2008, the European Union has been promoting different projects that aimed to revalue dredged port sediments, after a phytoremediation process, for agricultural use (food and ornamental crops) within the framework for the United Nations’ Sustainable Development Goals and the circular economy [13,14]. However, it is necessary to determine and study the real environmental impacts of these proposed alternatives.

One way to assess the relative environmental impact of different management strategies is life cycle assessment (LCA). LCA deals with the environmental aspects and impacts of a product system; for this purpose, it considers the complete life cycle of said product, from the extraction and acquisition of the raw material, through the production of energy and materials, the manufacture, the use and, finally, the end-of-life treatment and final disposal (cradle-to-grave perspective) [15]. According to Svensson et al. [16], the purpose of an LCA is to identify the step in the production chain where the environmental impact is greatest, to direct efforts toward this step, and focus on improving the weakest link in the production or treatment methodology, achieving an impact that is smaller and more sustainable.

In this context, the objective of this work was to analyze the environmental behavior of the use of phytoremediated port sediment as an alternative substrate to peat, focusing on lemon production (*Citrus limon* L. Burm) of the ‘Verna’ cultivar, grown under controlled experimental conditions using different proportions of port sediment. It should be noted that this work is presented as a continuity since the suitability of the port sediment as a culture substrate was previously confirmed, at the levels of both the vegetative development of the lemon trees and also the production and quality of its fruits [6,17].

## 2. Materials and Methods

The international standard used for the preparation of this work has been ISO 14040:2006 [15]; it has been divided into four phases: (i) definition of the objective and scope, (ii) inventory analysis, (iii) impact evaluation, and (iv) interpretation.

### 2.1. Study Product Definition

For the trials, port sediment mixed with a universal substrate was used as a culture medium. The universal substrate used was a commercial peat (Projar Professional, Valencia, Spain), while the port sediment came from the port of Livorno (Italy) and was previously phytoremediated over three years [18]. Three different culture media mixes were studied, based on their port sediment content, as shown in Table 1.

The cultivation of lemon trees (*Citrus limon* L. Burm var ‘Verna’) began in May 2020 with planting and ended in January 2022 when the lemon fruits were harvested. The trial was carried out in an experimental plot of Miguel Hernández University, located in Orihuela, southeast Spain (38°04′ N, 0°58′ W, 26 m above sea level). A total of 90 lemon trees were used (30 lemon trees × 3 culture media), planted in pots with a maximum capacity of 40 L (Table 1). Throughout the trial, water for the lemon trees was supplied by a drip irrigation system, with a different pipe for each culture media (25%, 50%, and 75% port sediment content) and a multi-outlet drip arch per tree (4 L h^−1^, Regaber, Matholding group, Barcelona, Spain). All the culture media were characterized before the lemon trees were planted and after the lemon fruits were harvested, aiming to identify both the main modifications caused during cultivation and the identification and definition of the control elements, due to their potential impact. The main results of the culture media characterization are shown in Table 2.

### 2.2. Objective and Scope

The objective of this study was to determine the environmental behavior with the use of phytoremediated port sediment as an alternative substrate to peat, focusing on the production of lemons (*Citrus limon* L. Burm var. ‘Verna’), grown under controlled experimental conditions with different proportions of phytoremediated port sediment (Table 1). This study is part of the LIFE SUBSED European Project (LIFE17 ENV/IT/347) whose main motivation is to contribute to the political debate on the recategorization and revaluation of port sediments, currently considered to be waste, by providing those responsible for making such decisions with objective and consolidated data.

According to ISO 14040, the scope must be sufficiently well defined to ensure that the breadth, depth, and level of detail of the study are compatible and sufficient to achieve the stated objective [15]. The scope included the definition of the product system, the functional unit, and the limits of the system.

**Figure 1:**
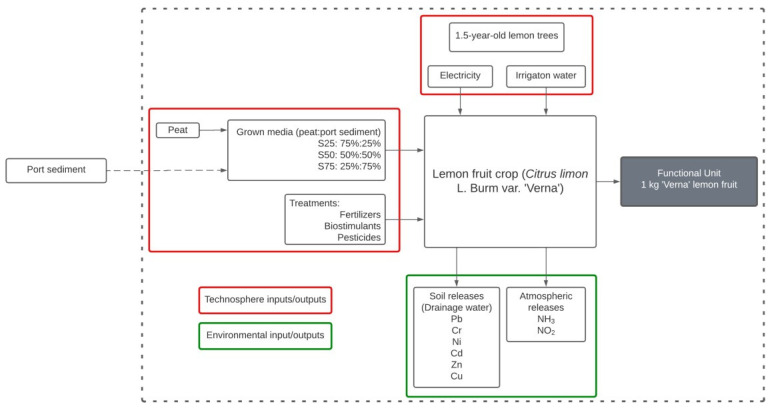
Scheme of the product system defined for this study.

We defined the ‘product system’ as the set of unit processes with elementary flows (inputs and outputs to the environment) and product flows (inputs and outputs to the technosphere) that performs one or more defined functions and that serves as a model for the cycle of life of a product [15]. The product system of this LCA is shown in Figure 1. The extraction and phytoremediation process of the dredged port sediment has been left out of the product system since it is of no interest in terms of the objectives of this study. The functional unit or reference unit used to measure the performance of the inputs and outputs of the product system corresponded to 1 kg of Verna lemons.

Delimiting the limits of an LCA is a very important step since it directly influences the results [15]. Considering that the objective of the study was focused on the environmental behavior of the port sediment as an agricultural substrate when applied to the lemon cultivation process, the limits of the system have been established from the cradle to the door of the experimental farm (cradle-to-gate). That is, the production of agricultural inputs has been considered (with the exception of the process of extraction and bioremediation of the dredged marine sediment), as well as the cultivation work needed until the lemons are harvested. Conversely, the processes that the lemons may undergo once they are harvested have not been considered, nor have their transport and distribution or the management of the organic waste that is generated. Nor has the impact produced by the occupation of the land and the transport of agricultural inputs to the experimental farm been considered, since they lack relevance to the comparative study of the substrate alternatives used.

### 2.3. Life Cycle Inventory Analysis

#### 2.3.1. Inventory Data

The inventory data have been obtained from the monitoring carried out on the experimental farm between May 2020 and January 2022, corresponding to the cultivation period (from planting the lemon trees to harvesting the lemons). The production data that were used constitute the real data of the number of fruits collected and their respective weight for each substrate alternative (Table 3). Both the correct vegetative development of the trees and the quality of the fruits obtained using the three types of substrates have been confirmed by previous, specific studies [6,17].

The addition of agricultural inputs (fertilizers, biostimulants, and pesticides) and networked water (drip irrigation system) was carried out according to the needs of the crop, keeping a rigorous record of the amounts added for each substrate alternative. Fertirrigation was systematized by TDR probes (Inta Crop Technology, Águilas, Spain), equipped with humidity, temperature, and electrical conductivity sensors, which made it possible to control the frequency of irrigation based on the weather forecast and crop needs.

Electricity was consumed exclusively by the fertigation system. In this way, the electrical consumption was calculated from the power demand of the fertigation equipment and the minutes of irrigation allocated for each substrate alternative.

The volume of drainage water (emission to the soil) was measured separately for each substrate alternative (Table 3). Additionally, on a laboratory scale, the concentrations of lead, chromium, nickel, cadmium, zinc, and copper present in the drainage water for each substrate alternative were quantified (Table 3). This allowed knowing the quantities of heavy metals emitted into the soil in each case (the result of multiplying the volume of drainage water by the concentration of each metal).

Air emissions were estimated using the procedure described by EMEP/EEA [19]. In this sense, the NH_3_ and NO_2_ air emissions were calculated from the amount of nitrogen applied in the form of fertilizer, according to Equation (1):

*E_pollutant_* = *AR_n_applied_* × *EF_pollutant_* (1)

where *E_pollutant_* represents the amount of pollutant emitted; *AR_n_applied_* represents the amount of nitrogen applied as fertilizer (kg); *EF_pollutant_* represents the emission factor of the pollutant (NH_3_= 0.05 kg kg ^−1^ of applied nitrogen and the NO_2_ = 0.04 kg kg^−1^ of applied nitrogen).

Table 4 presents the input and output data for matter, energy, and environmental flows, which are already normalized for the selected functional unit (1 kg of lemons). The data on the processes associated with the production of electricity, network water, fertilizers, biostimulants, and pesticides, as well as the extraction of peat, have been taken from the Ecoinvent 3 database [20]. Regarding the electricity demand, the process includes electricity produced in Spain and its transformation to medium voltage, as well as direct emissions into the air (SF6) and electricity losses during transmission. For water, the process considers existing water purification technologies in European territory. In the case of a commercial universal substrate (similar to peat in SimaPro), the process includes extraction and transport to the point of supply (average production in Europe). In the case of fertilizers (Isabion, ammonium nitrate, calcium nitrate, Novatec, and urea) the process includes manufacturing and transportation to the point of supply, based on the average European or global production, depending on the availability of the database. In the case of the biostimulant (Amalgerol), the process includes the collection of algae from the seabed, its processing, and the distribution of the usable product to a regional warehouse based on the average production in France. Finally, in the case of pesticides (abamectin, paraffin oil, and Doryoku), the process includes manufacturing and transportation to the point of supply, based on average production on a global scale. It should be noted that the activities associated with the preparation of the substrate (the mixing of the sediment and the peat) have not been taken into account, since they have been considered equivalent for all the substrate alternatives [21].

#### 2.3.2. Assignment Procedure

Most of the material and energy flows used in this LCA constitute specific primary data; that is, they are the actual data of the citrus cultivation process. Within this group are included: production data for lemons; peat consumption; consumption of agricultural inputs (fertilizers, biostimulants, and pesticides); consumption of irrigation water; electrical consumption; emissions to the soil of Pb, Cr, Ni, Cd, Zn, and Cu (drainage water).

A smaller amount of data constitutes the so-called secondary data, which have had to be calculated through estimates. This group includes the NH_3_ and NO_2_ emissions into the air. In any case, since all the data have been obtained in a disaggregated manner, it has not been necessary to apply rules for assigning or sharing the environmental loads.

#### 2.3.3. Sensitivity Analysis

A sensitivity analysis has been carried out on the estimated data for NH_3_ and NO_2_ emissions. Specifically, the results regarding how the LCA would alter if the NH_3_ and NO_2_ emissions were varied by ±20% were analyzed [22]. In this way, a new calculation of impacts has been made by changing these data; it has been established that the variation in the results of the life cycle impact assessment is minimal since emissions into the atmosphere do not have a substantial impact in comparison with the rest of the impacts.

### 2.4. Impact Assessment of the Life Cycle

The purpose of the Life Cycle Impact Assessment (LCIA) phase is to assess how significant the potential environmental impacts are, using the results of the LCI. Therefore, this phase is about assigning the data obtained in the ICV to the impact categories (classification stage), selecting an indicator for each of the categories later, and, finally, calculating the result of the indicator using a characterization factor (characterization stage). The classification and characterization stages are mandatory in an LCA. Optionally, it is possible to normalize (normalization stage) the results obtained in the characterization stage (which are expressed in heterogeneous units) with respect to a reference value to verify their relevance (obtaining the results in homogeneous units).

#### Evaluation Methodology

Among the evaluation methodologies, two large groups can be distinguished: the endpoint methodologies, oriented toward damage, and midpoint methodologies, oriented toward the environmental aspects. The methodology used in this report comes from the CML-IA baseline (version 4.7; 2016), developed by the Center of Environment Science (CML) of the University of Leiden (The Netherlands), which replaces the CML 2 baseline 2000. CML constitutes the reference midpoint methodology in terms of LCIA worldwide and is representative of the impacts generated in the life cycle of the use of the port sediment for the cultivation of lemon trees.

This methodology collects the impacts of the product life cycle in the following categories:

- Abiotic depletion: abiotic depletion potential (extraction of non-energy natural resources) in relation to the depletion potential of a reference resource (expressed in kg Sb-eq).
- Abiotic depletion (fossil fuels): potential depletion of fossil fuel reserves (extraction of energy resources) (expressed in MJ).
- Global warming (GWP100a): global warming potential for a time horizon of 100 years (expressed in kg CO_2_-eq).
- Ozone layer depletion (ODP): ozone layer depletion potential (expressed in kg CFC-11-eq).
- Human toxicity: toxicity to humans (expressed in kg 1.4-DB-eq).
- Fresh toilet aquatic ecotoxicity: ecotoxicity for freshwater resources (expressed in kg 1.4-DB-eq).
- Marine water ecotoxicity: ecotoxicity for the marine environment (expressed in kg 1.4-DB-eq).
- Terrestrial ecotoxicity: ecotoxicity for the terrestrial environment (expressed in kg 1.4- DB-eq).
- Photochemical oxidation: photochemical oxidation (expressed in kg C_2_H_4_-eq).
- Acidification: emission of acidifying gases into the atmosphere (expressed in kg SO_2_-eq).
- Eutrophication: eutrophication (expressed in kg PO_4_-eq).

The evaluation of the impacts generated by the life cycle of the citrus crop under this methodology has been developed with the SimaPro v. 9.3 software (PRé Sustainability B.V, Amersfoort, The Netherlands). SimaPro is a professional tool for calculating the LCA of products and services, developed by the Dutch company PRé Sustainability. SimaPro incorporates, in turn, access to the main databases of public and private characterization factors (Ecoinvent, Agrifootprint, ELCD, and Industry Data, among others) and the impact analysis methodologies used in the preparation of this LCA. The allocation system used in SimaPro has been used as the cut-off.

## 3. Results and Discussion

### 3.1. Interpretation of Inventory Data

In relation to the inventory analysis, the results confirmed that S50 (a mixture of 50% commercial universal substrate and 50% port sediment) provided the highest production of lemons (90.5 kg), followed by S25 (50.7 kg) and, finally, S75 (17.3 kg) (Table 3). Considering that S25 contained the lowest proportion of sediment (25%) and S75 the highest (75%), it seems reasonable to think that the cultivation of Verna lemons is affected when high proportions of port sediment are used in the growing medium; therefore, on the contrary, it presents good tolerance (in terms of higher production) when the intermediate proportions are used, as is the case with S25 and S50. The results are consistent with the results reported for other crops, such as strawberries and lettuce, where the authors indicated the negative impact of port sediment in high concentrations [23,24].

As can be seen in Table 4, the water demand, referenced to the functional unit (1 kg of lemons), was higher in the case of S75 (1698 kg water/kg lemons). This is because, despite the fact that, in absolute terms, the amount of irrigation water supplied was similar for all the culture media, the production was lower in the case of S75 (Table 4). On the contrary, S50 presented the lowest water consumption per kg of lemons produced, with a consumption of 329 kg water/kg lemons (Table 4), mainly due to both the higher production obtained in the trees grown with this substrate and the greater water retention caused by the characteristics of the port sediment, which has a more clayey structure, improving water retention and reducing its demand [18,25].

Along the same lines, given that electricity was consumed exclusively in the drip irrigation system, S50 had a lower consumption (0.629 kWh/kg lemons), followed by S25 (1.74 kWh/kg lemons) < S75 (3.33 kWh/kg lemons) as shown in Table 4.

Regarding drainage water, the largest volume of leachate in absolute terms was collected for S25 (6.9 m^3^), followed by S50 (4.2 m^3^), and S75 (3.4 m^3^). This would be reasonable since crops with higher sediment contents present greater compaction, so they would better retain water and, therefore, generate less leachate. However, if we express the volume of drainage water per kg of lemons produced, S50 presents a lower ratio (0.05 m^3^/kg lemons), followed by S25 (0.14 m^3^/kg lemons), and S75 (0.20 m^3^/kg lemons). Consequently, S50 deposited a lower amount of metal emissions into the soil per kg of lemons obtained (Table 4).

Atmospheric emissions were estimated from the amount of nitrogen applied in the form of fertilizer, so they followed the same path as the latter. Thus, both NH_3_ and NO_2_ emissions per kg of lemons were lower in the case of S50 (0.440 g/kg for NH_3_ and 0.352 g/kg for NO_2_), followed by S25 (0.896 g/kg for NO_2_). NH_3_ and 0.717 g/kg for NO_2_) were < (2.27 g/kg for NH_3_ and 1.82 g/kg for NO_2_) (Table 4).

### 3.2. Interpretation of Life Cycle Impact Assessment

**Figure 2:**
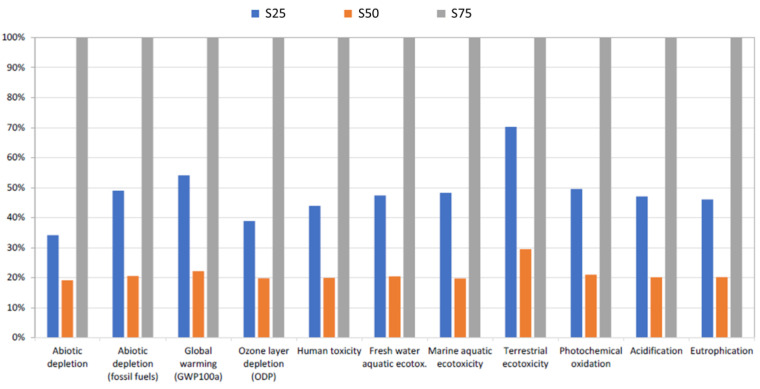
Comparison of the life cycle impact of 1 kg of lemons for the three substrate alternatives, using the CML-IA baseline methodology, showing the characterization stage. Relativized values are shown as a percentage with respect to the greatest impact recorded in each category.

Table 5 shows the comparison of the life cycle impact (characterization stage) of 1 kg of lemons for the three substrate alternatives studied. The data are shown in absolute terms, and it can be seen that S75 was the sample with the greatest impact for all the categories included in the methodology. To facilitate understanding, Figure 2 shows these same data relativized as a percentage with respect to the greatest impact recorded in each category, which corresponds to S75 in all cases. In this way, the impacts associated with S50 are approximately 80% lower than those associated with S75 for all impact categories, except in terms of the ecotoxicity of the terrestrial environment, which is somewhat lower (71% lower). In summary, the lower production obtained in S75 translated into higher environmental impacts per kg of lemons.

The units used in the characterization stage are heterogeneous (that is, each impact category has its own units); therefore, if you want to compare the impact derived from the production of 1 kg of lemons with the different categories, it is convenient to normalize the results (conversion to homogeneous units). The normalization was carried out using the normalization factors set by the CML-IA baseline methodology for the European Union. The results of the normalization stage made it possible to identify the categories whose contribution to the global impact was greater. Thus, the most affected categories were ecotoxicity for the marine environment, abiotic depletion potential, ecotoxicity to freshwater resources, and toxicity to humans.

**Figure 3:**
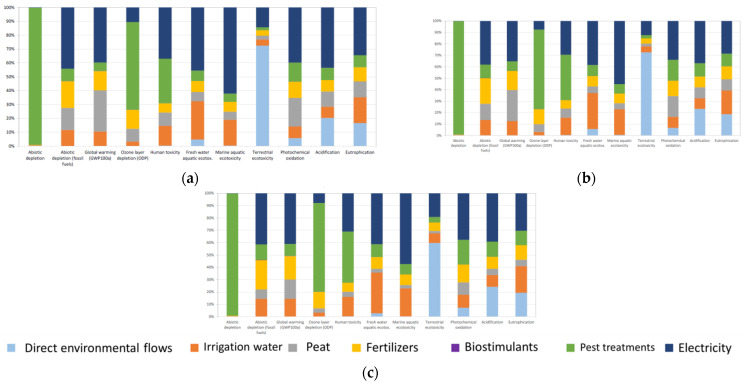
Characterization stage of the impacts of 1 kg of lemons for a specified methodology (CML-IA baseline) for S25 (**a**); S50 (**b**); and S75 (**c**) culture media. Relative values. Direct environmental flows (elementary flows) represent atmospheric emissions (NH_3_ and NO_2_) and discharges to the ground (metals contained in drainage water). Long-term emissions (>100 years) are included, and the infrastructure processes are excluded.

The contribution in absolute terms of each of the processes contemplated in this LCA (direct environmental flows (emissions to the air and to the soil), production of network water, peat production, fertilizer production, biostimulant production, pesticide production, and electricity production) on the different impact categories are shown in Table 6, Table 7 and Table 8, where the results for S25, S50, and S75, respectively, are shown. To facilitate understanding, the results are also presented in relative terms (Figure 3). At first glance, it can be seen that the contribution pattern is similar for the three substrate alternatives, so the explanation that follows has focused exclusively on S50, since it has a lower impact, and on the impact categories that were more affected than those that were detected in the normalization stage:

- Regarding ecotoxicity for the marine environment, the processes that contributed the most to this category were the production of electricity (55.1%), mains water (22.3%), fertilizers (8.5%), and pesticides (8.2%).
- Regarding the abiotic depletion potential, the production of fertilizers (99.0%) was the largest contributor to this category. This is due to the large number of raw materials (chemicals and minerals) used in the manufacture of pesticides.
- Regarding ecotoxicity for freshwater resources, the processes that contributed the most to this category were electricity production (38.4%), irrigation water (31.4%), pesticides (9.7%), and fertilizers (9.1%).
- Regarding toxicity to humans, the processes that contributed the most to this category were the production of pesticides (39.7%), electricity (29.5%), and tap water (15.3%).

Finally, the main impacts derived from the direct environmental flows were generated in terms of terrestrial ecotoxicity (72.7%), atmospheric acidification (23.3%), and water eutrophication (18.6%) (Figure 3). These impacts are produced as a result of the atmospheric emissions of NH_3_ and NO_2_ derived from the use of nitrogenous fertilizers and metal emissions to the soil together with drainage water. In any case, its lower impact is notable compared to the processes that take place upstream (electricity production, network water, fertilizers, biostimulants, and pesticides). This reflects the importance of influencing not only the reduction of the direct environmental impacts generated in the production of Verna lemons but also in the entire chain of the product’s life cycle.

## 4. Conclusions

The results of the study confirmed that the environmental footprint of the use of port sediment for the cultivation of ‘Verna’ lemon trees increases as its percentage in the culture medium increasses. This is mainly due to its effect on lemon yields. In this way, the lower yields of lemons obtained from lemon trees grown in the substrate with port sediment (S75) translated into greater environmental impacts per kilo of fruit.

In this context, and based on the results, the potential for the use of port sediments mixed with other substrates was confirmed, employing media mixes that do not exceed 50% port sediment, in order to maintain optimum fruit yield and quality, increase water retention, thereby reducing its consumption, and ultimately reduce the environmental impacts of port sediments.

## Figures and Tables

**Table 1:** Port sediment and peat, mixed in the culture media used in this study. The values represent the substrate content.

Peat Content			Port Sediment Content	
Acronym	(L per 40 L pot)		(L per 40 L pot)	
		(%)		(%)
S25	30	75	10	25
S50	20	50	20	50
S75	10	25	30	75

Values expressed in L for each 40 L pot and in volume (%).

**Table 2:** Initial characterization (before planting, May 2020) and final characterization (after harvesting the lemons, January 2022) of the culture media used in this study.

	Hydrocarbons C10–C14	Lead (Pb)	Cadmium (Cd)	Nickel (Ni)	Chromium (Cr)	Mercury (Hg)	Copper (Cu)	Zinc (Zn)
	(µg kg^−1^)				(mg kg^−^^1^)			
Initial-Before lemon tree planting (May 2020)								
S25	40.0	38.7	0.8	34.1	44.5	0.1	29.0	150
S50	60.9	44.6	0.9	57.0	68.0	0.2	38.4	228
S75	17.8	50.3	1.4	59.0	70.0	0.1	41.9	245
Final-After lemon fruit harvest (January 2022)								
S25	0.0	48.9	0.9	52.0	54.0	0.1	37.8	485
S50	0.0	58.7	1.1	61.0	69.0	0.2	47.8	352
S75	0.0	57.2	1.1	61.0	79.0	0.2	49.9	296

**Table 3:** Experimental data used in the LCA study of port sediment as a culture media for ‘Verna’ lemon cultivation.

Culture Media			
Parameter	S25	S50	S75
Lemon fruits production			
Number of fruits	290	590	124
Weight (kg)	50.7	90.5	17.3
Drainage water			
Drainage volume (m^3^) *	0.14	0.05	0.20
Pb (µg L^−^^1^)	5.22	0.0	0.0
Cr (µg L^−^^1^)	11.1	0.0	0.0
Cd (µg L^−^^1^)	22.73	40.03	13.24
Zn (µg L^−^^1^)	603	607.3	533.4
Cu (µg L^−^^1^)	63.6	101.2	67.27

* Drainage volume obtained for the production of 1 kg of lemons (functional unit).

**Table 4:** Input and output inventory for the production of 1 kg of ‘Verna‘ lemons, cultivated on different culture media, based on a port sediment and peat mix.

Culture Media					
	Parameter	Unit	S25	S50	S75
	Electricity	kWh	1.74	0.629	3.33
	Irrigation water	kg	673	329	1698
	Peat	kg	33.7	12.6	33.0
	Isabión (10% Syngenta)	g	18.5	9.03	46.6
Inputs	Amalgerol	g	18.9	9.24	47.6
	Ammonium nitrate	g	13.4	6.54	33.7
	Calcium nitrate	g	31.7	15.5	79.8
	Novatec (20-5-10)	g	32.1	15.7	81.0
	Pest treatments	g	9.21	5.16	27.0
	Urea	g	0.985	0.552	2.89
	Pb to soil	mg	0.705	0.00	0.00
	Cr to soil	mg	1.50	0.00	0.00
	Ni to soil	mg	3.07	1.88	2.59
Outputs	Cd to soil	mg	0.397	0.141	0.511
	Zn to soil	mg	81.4	28.5	104
	Cu to soil	mg	8.59	4.75	13.2
	NH_3_ to air	g	0.896	0.440	2.27
	NO_2_ to air	g	0.717	0.352	1.82

**Table 5:** Comparison of the life cycle impact of 1 kg of lemons for the three substrate alternatives, with different proportions of port sediment.

Culture Media				
Impact Category	Unit	S25	S50	S75
Abiotic depletion	kg Sb eq	1.58 × 10^−^^5^	8.83 × 10^−^^6^	4.62 × 10^−^^5^
Abiotic depletion (fossil fuels)	MJ	1.38 × 10^+1^	5.80	2.82 × 10^+1^
Global warming (GWP100a)	kg CO_2_ eq	1.33	5.45 × 10^−^^1^	2.47
Ozone layer depletion (ODP)	kg CFC-11 eq	2.59 × 10^−^^7^	1.32 × 10^−^^7^	6.67 × 10^−^^7^
Human toxicity	kg 1.4-DB eq	4.42 × 10^−^^1^	2.01 × 10^−^^1^	1.01
Fresh water aquatic ecotoxicity	kg 1.4-DB eq	3.03 × 10^−^^1^	1.30 × 10^−^^1^	6.40 × 10^−^^1^
Marine aquatic ecotoxicity	kg 1.4-DB eq	1.29 × 10^+3^	5.26 × 10^+2^	2.67 × 10^+3^
Terrestrial ecotoxicity	kg 1.4-DB eq	4.07 × 10^−^^3^	1.71 × 10^−^^3^	5.80 × 10^−^^3^
Photochemical oxidation	kg C_2_H_4_ eq	3.47 × 10^−^^4^	1.47 × 10^−^^4^	7.01 × 10^−^^4^
Acidification	kg SO_2_ eq	8.84 × 10^−^^3^	3.78 × 10^−^^3^	1.8 × 10^−^^2^
Eutrophication	kg PO_4_--- eq	2.45 × 10^−^^3^	1.08 × 10^−^^3^	5.3 × 10^−^^3^

CML-IA baseline methodology. Characterization stage. Absolute values.

**Table 6:** S25 Characterization stage of the impacts of 1 kg of lemons for the specified methodology (CML-IA baseline).

Impact Category	Unit	Direct Environmental Flows	Irrigation Water	Peat	Fertilizers	Bio Stimulant	Pest Treatment	Electricity
Abiotic depletion	kg Sb eq	0.00	6.95 × 10^−8^	1.8 × 10^−8^	6.59 × 10^−8^	2.46 × 10^−11^	1.56 × 10^−5^	3.7 × 10^−8^
Abiotic depletion (fossil fuels)	MJ	0.00	1.61	2.19	2.65	2.48 × 10^−2^	1.21	6.09
Global warming (GWP100a)	kg CO_2_ eq	0.00	1.41 × 10^−1^	3.95 × 10^−1^	1.85 × 10^−1^	1.45 × 10^−3^	8.15 × 10^−2^	5.30 × 10^−1^
Ozone layer depletion (ODP)	kg CFC- 11 eq	0.00	8.49 × 10^−9^	2.39 × 10^−8^	3.54 × 10^−8^	2.0 × 10^−10^	1.63 × 10^−7^	2.73 × 10^−8^
Human toxicity	kg 1.4- DB eq	1.84 × 10^−3^	6.27 × 10^−2^	4.23 × 10^−2^	2.97 × 10^−2^	3.8 × 10^−5^	1.42 × 10^−1^	1.63 × 10^−1^
Fresh water aquatic ecotoxicity	kg 1.4- DB eq	1.45 × 10^−2^	8.37 × 10^−2^	2.03 × 10^−2^	2.42 × 10^−2^	1.49 × 10^−5^	2.25 × 10^−2^	1.38 × 10^−1^
Marine aquatic ecotoxicity	kg 1.4- DB eq	52.6	2.40 × 10^+2^	7.43 × 10^+1^	9.16 × 10^+1^	4.9 × 10^−2^	7.70 × 10^+1^	8.01 × 10^+2^
Terrestrial ecotoxicity	kg 1.4- DB eq	2.95 × 10^−3^	1.79 × 10^−4^	1.13 × 10^−4^	1.59 × 10^−4^	1.21 × 10^−7^	8.72 × 10^−5^	5.82 × 10^−4^
Photochemical oxidation	kg C_2_H_4_ eq	2.02 × 10^−5^	2.91 × 10^−5^	7.16 × 10^−5^	4.05 × 10^−5^	9.08 × 10^−8^	4.7 × 10^−5^	1.38 × 10^−4^
Acidification	kg SO_2_ eq	1.80 × 10^−3^	7.15 × 10^−4^	9.70 × 10^−4^	7.23 × 10^−4^	1.08 × 10^−6^	7.82 × 10^−4^	3.85 × 10^−3^
Eutrophication	kg PO_4_- -- eq	4.09 × 10^−4^	4.56 × 10^−4^	2.83 × 10^−4^	2.49 × 10^−4^	1.93 × 10^−7^	2.12 × 10^−4^	8.46 × 10^−4^

**Table 7:** S50 Characterization stage of the impacts of 1 kg of lemons for the specified methodology (CML-IA baseline). Absolute values. Long-term emissions (>100 years) are included, and infrastructure processes are excluded.

Impact Category	Unit	Direct Environmental Flows	Irrigation Water	Peat	Fertilizers	Biostimulant	Pest Treatment	Electricity
Abiotic depletion	kg Sb eq	0.00	3.40 × 10^−8^	7.03 × 10^−9^	3.23 × 10^−8^	1.21 × 10^−11^	8.74 × 10^−6^	1.37 × 10^−8^
Abiotic depletion (fossil fuels)	MJ	0.00	7.87 × 10^−1^	8.19 × 10^−1^	1.30	1.21 × 10^−2^	6.7 × 10^−1^	2.20
Global warming (GWP100a)	kg CO_2_ eq	0.00	6.90 × 10^−2^	1.48 × 10^−1^	9.07 × 10^−2^	7.1 × 10^−4^	4.57 × 10^−2^	1.92 × 10^−1^
Ozone layer de- pletion (ODP)	kg CFC- 11 eq	0.00	4.15 × 10^−9^	8.94 × 10^−9^	1.73 × 10^−8^	9.7 × 10^−11^	9.16 × 10^−8^	9.8 × 10^−9^
Human toxicity	kg 1.4- DB eq	8.64 × 10^−04^	3.07 × 10^−2^	1.58 × 10^−2^	1.45 × 10^−2^	1.90 × 10^−5^	7.96 × 10^−2^	5.92 × 10^−2^
Fresh water aquatic ecotoxicity	kg 1.4- DB eq	7.47 × 10^−03^	4.09 × 10^−2^	7.57 × 10^−3^	1.18 × 10^−2^	7.29 × 10^−6^	1.26 × 10^−2^	5.0 × 10^−2^
Marine aquatic ecotoxicity	kg 1.4- DB eq	2.99	1.18 × 10^+2^	2.78 × 10^+1^	4.48 × 10^+1^	2.4 × 10^−2^	4.31 × 10^+1^	2.90 × 10^+2^
Terrestrial ecotoxicity	kg 1.4- DB eq	1.24 × 10^−03^	8.76 × 10^−5^	4.2 × 10^−5^	7.76 × 10^−5^	5.90 × 10^−8^	4.89 × 10^−5^	2.1 × 10^−4^
Photochemical oxidation	kg C_2_H_4_ eq	9.80 × 10^−06^	1.42 × 10^−5^	2.68 × 10^−5^	1.98 × 10^−5^	4.4 × 10^−8^	2.68 × 10^−5^	5.0 × 10^−5^
Acidification	kg SO_2_ eq	8.79 × 10^−04^	3.49 × 10^−4^	3.63 × 10^−4^	3.54 × 10^−4^	5.29 × 10^−7^	4.38 × 10^−4^	1.39 × 10^−3^
Eutrophication	kg PO_4_- eq	2.0 × 10^−04^	2.23 × 10^−4^	1.06 × 10^−4^	1.2 × 10^−4^	9.4 × 10^−8^	1.19 × 10^−4^	3.06 × 10^−4^

**Table 8:** S75 Characterization stage of the impacts of 1 kg of lemons for the specified methodology (CML-IA baseline). Absolute values. Long-term emissions (>100 years) are included, and infrastructure processes are excluded.

Impact Category	Unit	Direct Environmental Flows	Irrigation Water	Peat	Fertilizers	Biostimulant	Pest Treatment	Electricity
Abiotic depletion	kg Sb eq	0.00	1.75 × 10^−7^	1.84 × 10^−8^	1.6 × 10^−7^	6.2 × 10^−11^	4.58 × 10^−5^	7.2 × 10^−8^
Abiotic depletion (fossil fuels)	MJ	0.00	4.06	2.14	6.69	6.25 × 10^−2^	3.55	1.17 × 10^+1^
Global warming (GWP100a)	kg CO_2_ eq	0.00	3.56 × 10^−1^	3.87 × 10^−1^	4.68 × 10^−1^	3.67 × 10^−3^	2.39 × 10^−1^	1.01
Ozone layer depletion (ODP)	kg CFC- 11 eq	0.00	2.14 × 10^−8^	2.34 × 10^−8^	8.94 × 10^−8^	5.04 × 10^−10^	4.80 × 10^−7^	5.2 × 10^−8^
Human toxicity	kg 1.4- DB eq	3.02 × 10^−3^	1.58 × 10^−1^	4.14 × 10^−2^	7.50 × 10^−2^	9.79 × 10^−5^	4.17 × 10^−1^	3.13 × 10^−1^
Fresh water aquatic ecotoxicity	kg 1.4- DB eq	1.76 × 10^−2^	2.1 × 10^−1^	1.98 × 10^−2^	6.10 × 10^−2^	3.76 × 10^−5^	6.62 × 10^−2^	2.64 × 10^−1^
Marine aquatic ecotoxicity	kg 1.4- DB eq	5.42	6.06 × 10^+2^	7.28 × 10^+1^	2.31 × 10^+2^	1.26 × 10^−1^	2.26 × 10^+2^	1.53 × 10^+3^
Terrestrial ecotoxicity	kg 1.4- DB eq	3.46 × 10^−3^	4.52 × 10^−4^	1.1 × 10^−4^	4.0 × 10^−4^	3.04 × 10^−7^	2.56 × 10^−4^	1.1 × 10^−3^
Photochemical oxidation	kg C_2_H_4_ eq	5.10 × 10^−5^	7.34 × 10^−5^	7.02 × 10^−5^	1.02 × 10^−4^	2.29 × 10^−7^	1.40 × 10^−4^	2.64 × 10^−4^
Acidification	kg SO_2_ eq	4.54 × 10^−3^	1.80 × 10^−3^	9.49 × 10^−4^	1.82 × 10^−3^	2.73 × 10^−6^	2.30 × 10^−3^	7.36 × 10^−3^
Eutrophication	kg PO_4_- eq	1.03 × 10^−3^	1.15 × 10^−3^	2.7 × 10^−4^	6.28 × 10^−4^	4.87 × 10^−7^	6.2 × 10^−4^	1.62 × 10^−3^

## Data Availability

Data are contained within the article.
